# Interface Adsorption versus Bulk Micellization of Surfactants: Insights from Molecular Simulations

**DOI:** 10.1021/acs.jctc.3c00223

**Published:** 2023-05-22

**Authors:** Matej Kanduč, Cosima Stubenrauch, Reinhard Miller, Emanuel Schneck

**Affiliations:** † Jožef Stefan Institute, Jamova 39, 1000 Ljubljana, Slovenia; ‡ Institute of Physical Chemistry, University of Stuttgart, Pfaffenwaldring 55, 70569 Stuttgart, Germany; ¶ Department of Physics, Technische Universität Darmstadt, Hochschulstrasse 8, 64289 Darmstadt, Germany

## Abstract

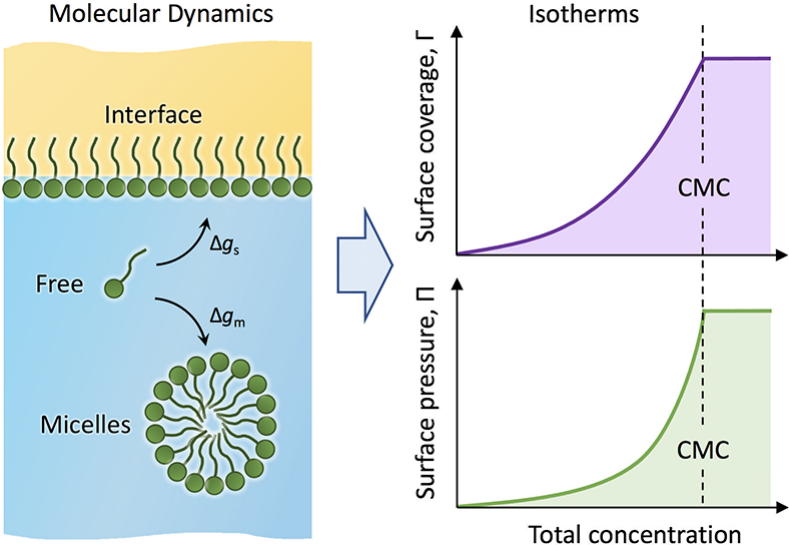

Surfactants play essential roles in many commonplace applications and industrial processes. Although significant progress has been made over the past decades with regard to model-based predictions of the behavior of surfactants, important challenges have remained. Notably, the characteristic time scales of surfactant exchange among micelles, interfaces, and the bulk solution typically exceed the time scales currently accessible with atomistic molecular dynamics (MD) simulations. Here, we circumvent this problem by introducing a framework that combines the general thermodynamic principles of self-assembly and interfacial adsorption with atomistic MD simulations. This approach provides a full thermodynamic description based on equal chemical potentials and connects the surfactant bulk concentration, the experimental control parameter, with the surfactant surface density, the suitable control parameter in MD simulations. Self-consistency is demonstrated for the nonionic surfactant C_12_EO_6_ (hexaethylene glycol monododecyl ether) at an alkane/water interface, for which the adsorption and pressure isotherms are computed. The agreement between the simulation results and experiments is semiquantitative. A detailed analysis reveals that the used atomistic model captures well the interactions between surfactants at the interface but less so their adsorption affinities to the interface and incorporation into micelles. Based on a comparison with other recent studies that pursued similar modeling challenges, we conclude that the current atomistic models systematically overestimate the surfactant affinities to aggregates, which calls for improved models in the future.

## 1 Introduction

Surfactants (or “surface-active agents”) have a polar headgroup compatible with water and a nonpolar tail compatible with oil. This dual nature lends surfactants their unique ability of self-assembly in aqueous solutions and adsorption to various interfaces. Surfactants are paramount to a wide range of technological applications—ranging from everyday home and personal care products all the way to agriculture, petrochemistry, and beyond. Thus, it is no surprise that much of contemporary soft matter research is driven by a quest to understand and control surfactant self-assembly from fundamental and technological perspectives.^1^ Indeed, self-assembly phenomena have been in sharp focus of research for about a century.^2^ Practically all scientific methods have been used to study surfactants—ranging from experiments and theoretical calculations to computer simulations.^3^

Simulations have developed tremendously over the past several decades and nowadays represent one of the core pillars for the exploration of phenomena and processes involving surfactants. Atomistic molecular dynamics (MD) simulations are a valuable tool, as they can discriminate among the chemical characteristics of amphiphilic compounds and capture the basic driving forces, such as hydrophobic and hydration interactions.^2^ Moreover, MD simulations can provide insights into microscopic details, some of which are experimentally difficult to access, such as adsorption densities and conformational structures of surfactants. There are, however, challenges that have limited the practical usefulness of MD simulations in a range of important cases, as will be explained in the following.

Self-assembled structures, in general, must be viewed as dynamic, with the surfactant molecules constantly exchanging between the aggregate or interface and the bulk solution. This diffusive exchange can be understood as a thermally activated process in which the molecule has to surmount the free energy barrier to escape from the aggregate or interface into the bulk. The barrier scales roughly linearly with the alkyl chain length,^4,5^ therefore, the exchange time increases exponentially with the chain length—by about a factor of 2–3 for each CH_2_ group added to the chain.^6^

**Figure 1:**
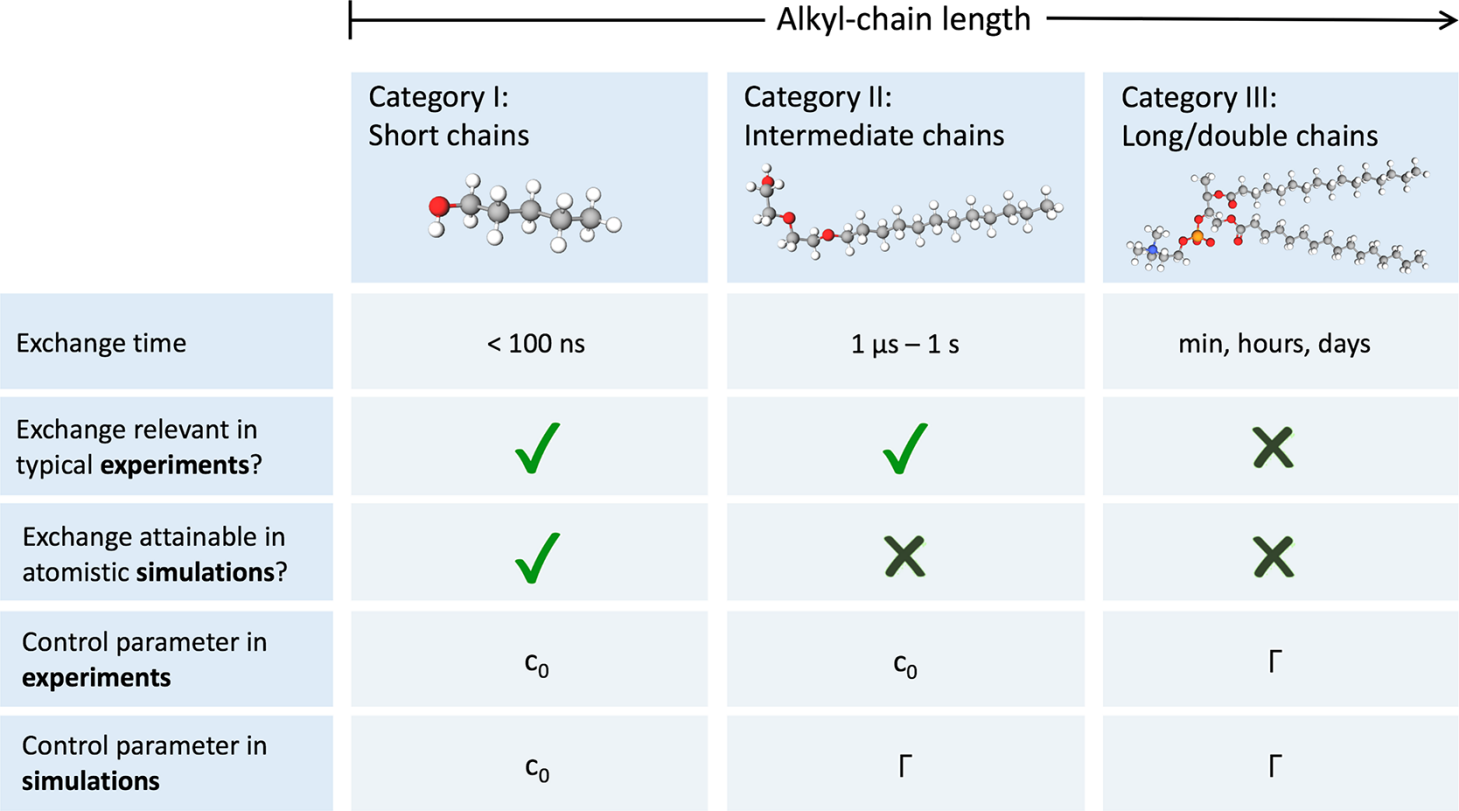
Categorization of surfactants based on the time scale for their exchange between bulk and interfaces or aggregates, which primarily depends on their alkyl-tail length. The exchange time scale further governs the adequate boundary condition and the relevant control parameter of an interface, which can be either the total concentration of surfactants (*c*_0_) or the surface coverage (Γ). Category I consists of very short-chained surfactants whose exchange is rapid enough to be followed in atomistic MD simulations. The relevant control parameter in this case is the total concentration. For surfactants of category II with intermediate chain lengths, the exchange is too slow to be captured by simulations but rapid on the time scale of most experiments. Long- and double-chained lipids belonging to category III do not exchange between the bulk and interface both on experimental and simulation time scales. In this case, the relevant parameter is surface excess Γ.

In atomistic MD simulations, typical systems nowadays contain up to 10^6^ atoms while the typical time scales reach up to a few (tens of) μs, which brings us to a critical time scale categorization of surfactant modeling, as outlined in Figure 1. Category I comprises short-chain surfactants (with chain lengths roughly up to 5–7 carbon atoms), which feature high solubility (sometimes not even forming micelles). They form loose monolayers and exhibit rapid exchange between the interface and the bulk (on the scale of nanoseconds). In atomistic simulations, their adsorption equilibria can readily be monitored directly by letting the system explore its kinetic pathway during the aggregation or adsorption of individual molecules.^5,7−10^ As a consequence, the relevant control parameter in both simulations and experiments for this category is the total number of surfactants in the system, also expressed as the total concentration, *c*_0_.^11^

The other side of the spectrum, category III, consists of long-chained (typically ≳ C18) or double-chained surfactants (also called “lipids”) that exchange so slowly with the bulk that the exchange is not witnessed even on the experimental time scales. Because of their slow dynamics and very low solubility, they are referred to also as insoluble surfactants. They form essentially water-insoluble (Langmuir-type) monolayers, for which the exchange with the bulk can be neglected.^12−14^ The relevant control parameter for both experiments and simulations is then the areal density of adsorbed surfactants, described by the surface excess Γ.

Finally, surfactants from category II, featuring alkyl chains of intermediate length (mainly C7–C16), adsorb as Gibbs monolayers and exchange on time scales of microseconds to seconds, which is too slow to be captured in simulations but rapid in comparison to the experimental time scale. This means that the relevant experimental control parameter is the bulk concentration *c*_0_, whereas the control parameter in simulations is the surface excess Γ, see Figure 1. In other words, there is a mismatch between what is observed in experiments and what in simulations. To make matters even worse, the required simulation time should be much longer than the exchange time to sample the dynamic equilibrium adequately. For this reason, category II represents the greatest modeling challenge. This is particularly unfortunate because the most frequently used surfactants in technological applications belong to category II, with tail lengths revolving around C12 as a common compromise between the surface activity and the adsorption time.^15^

To date, atomistic MD simulation studies of surfactants from category II have mainly been used to investigate specific aspects of adsorption layers with defined Γ, where exchange between surface and bulk is irrelevant. These aspects include molecular conformations, interactions, and the equation of state (EoS), i.e., the relation between Γ and the surface pressure Π.^16−21^ Early works have identified the influence of surfactant chemistry on the surface tension at fixed Γ,^16,17^ while later studies revealed surfactant clustering at the air/water interface,^18,19^ which was found to be largely suppressed at oil/water interfaces.^21^ In order to capture with MD simulations also those aspects that involve surfactant exchange between interface and bulk, one needs to resort to advanced computational techniques that take the surfactant chemical potential into account. Progress in this direction has been made only rather recently. A study by Huston and Larson^22^ investigated the adsorption of poly(ethylene glycol) oligomers and polyethylene glycol alkyl ethers at water/alkane interfaces. Another study combined the adsorption free energy to the bare air/water interface with the EoS to predict the adsorption and surface pressure isotherms, Γ(*c*_0_) and Π(*c*_0_), respectively.^11^ A very recent study reported the surface activity of polyethoxylated alkyl ether surfactants for alkane/water interfaces as a function of the headgroup length.^23^

Here, we develop a full thermodynamic description of surfactant solutions in contact with an interface. The connection between the three different states in which the surfactants can exist—as free surfactants, as micelles, and adsorbed to the interface—is drawn rigorously on the basis of equal surfactant chemical potentials, which we determine in atomistic MD simulations with the help of free-energy calculations. We showcase our method for the hexaethylene glycol monododecyl ether C_12_EO_6_ in an aqueous phase and at an alkane/water interface. The approach enables us to extract not only the EoS from the simulation model but also the adsorption and pressure isotherms, which can be directly compared to experimental data. Despite the enormous recent progress in molecular modeling,^11,22−24^ to the best of our knowledge, this study is the first that provides a complete self-consistent thermodynamic description of surfactant adsorption and micellization based on an atomistic model.

## 2 Thermodynamic Principles

### 2.1 Chemical Equilibrium between Different Phases

**Figure 2:**
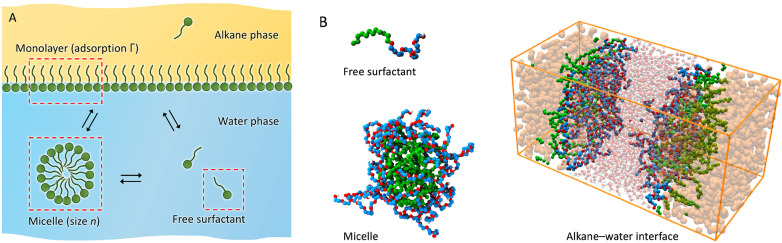
(A) Schematic presentation of the studied system, featuring a monolayer phase, bulk water with free surfactants, and micelles. The surfactant exchange with the bulk alkane phase is not in our scope. Computer simulations are performed on distinct phases, highlighted by red dashed rectangles. (B) Representative snapshots of the three simulated phases: A free surfactant C_12_EO_6_ (with a green alkyl united-atom tail and a red–blue EO head) in water, a micelle in water (water not shown for clarity), and the simulation box of water and alkane slabs with adsorbed surfactants at both interfaces.

Atomistic MD simulations do not currently enable modeling the exchange of surfactants between interfaces or aggregates and the aqueous bulk. One reason is that a prohibitively large bulk volume would have to be simulated to represent the low surfactant concentrations that are thermodynamically relevant to observe the adsorption or aggregation equilibria. Another reason, as emphasized in the introduction, is the inaccessible time scales of the exchange. Our strategy is, therefore, to model small fractions (<10 nm in size) of different phases separately and connect them via thermodynamic principles. The decisive phases for our description are the bulk solution of free surfactants, the micelle phase, and the monolayer of adsorbed surfactants at the interface, as schematically depicted in Figure 2A. Representative simulation snapshots of the three phases are shown in Figure 2B.

The chemical potentials of surfactants in the three phases can be written as
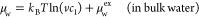
1
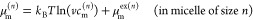
2
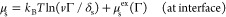
3The first term in each equation corresponds to the ideal chemical potential, expressed by the surfactant concentration in a given phase. With *c*_1_ we denote the concentration of individual (free) surfactants in the aqueous bulk, and *c*_m_^(*n*)^ is the concentration of surfactants in the micelle of size (i.e., aggregation number) *n*. The adsorbed surfactant monolayer at the interface is characterized by the excess adsorption Γ, corresponding to the areal density of surfactants. Its volume concentration is Γ/δ_s_, where δ_s_ is the effective thickness of the attractive interface potential. Finally, *v* is the normalizing coefficient, arbitrarily chosen as the volume of the surfactant.

The second term in the above equations stands for the excess chemical potential. While μ_m_^ex(*n*)^ depends on micelle size *n* and μ_s_^ex^ on interface adsorption Γ, the excess chemical potential in water μ_w_^ex^ will be considered a constant, that is, independent of the concentration *c*_1_. We will later justify this ideal-solution assumption by showing that possible concentration values *c*_1_ are small enough. In thermal equilibrium, all three chemical potentials match, μ_w_ = μ_m_^(*n*)^ = μ_s_(Γ), which will help us to obtain a full thermodynamic description of the monolayer and micellar environment at a given surfactant concentration.

### 2.2 Thermodynamics of Micelle Self-Assembly

We now outline the fundamental thermodynamic principles of surfactant self-assembly into micelles.^25,26^ Consider a reaction in which a free surfactant combines with a micelle of size *n* – 1, resulting in a micelle of size *n*. The Gibbs free energy change for this process is , where  and  are the chemical potentials of the micelles of sizes *n* and *n* – 1, respectively, and μ_w_ is the chemical potential of free surfactants in the bulk water phase. With the tilde sign, we distinguish the chemical potentials of micelles from the chemical potentials of individual surfactants. In thermodynamic equilibrium, the law of mass action demands that the Gibbs free energy change when going either direction of the reaction be zero, Δ*G* = 0, which implies
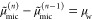
4The chemical potentials of micelles are composed of the ideal and excess parts, , where *c*_*n*_ is the concentration of micelles of size *n*. Furthermore, the difference between the excess parts of the micelles of sizes *n* and *n* – 1 is the excess chemical potential of one surfactant in the micelle of size *n*, that is, . With these considerations, the above equation becomes
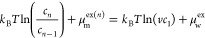
5Moreover, the difference in the excess chemical potential of a surfactant in the micelle and in water represents the transfer free energy for bringing a surfactant from bulk water into the micelle of final size *n*, which we express as Δ*g*_m_^(*n*)^ = μ_m_^ex(*n*)^ – μ_w_^ex^. This finally brings us to the following well-known iterative expression for micelle concentrations^6^
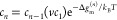
6This equation represents a complete description of the micelle-size distribution based on the concentration of free surfactants, *c*_1_, provided that the transfer free energies Δ*g*_m_^(*n*)^ are known—the main technical challenge of this study. Note that this thermodynamic framework assumes micelles as independent entities in solution, which do not interact with each other. This simplification would, however, break down only at very high concentrations.

From known concentrations and the conservation of the total number of surfactants, we define the *total concentration* as
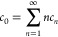
7The total concentration is the primary experimentally controlled parameter for categories I and II (Figure 1), defined as the overall amount of surfactant added to the system. Therefore, it is practical to express the final simulations result in terms of *c*_0_. We will connect *c*_0_ to *c*_1_ further below.

### 2.3 Thermodynamics of Monolayer Formation

In general, Gibbs monolayers at various interfaces can be rather complex, featuring a two-phase or even multiphase coexistence—a 2D analog to the 3D micellization in bulk. The simplest possible scenario is a uniform monolayer for all coverages Γ. Such a monolayer forms at oil/water interfaces with C_12_EO_6_ surfactants, as used in this study. A uniform monolayer at the interface is, therefore, fully characterized solely by the excess adsorption Γ.

In thermodynamic equilibrium, the surfactants in the adsorbed monolayer have the same chemical potential, μ_s_, as free surfactants in the bulk water phase, μ_w_. Equating eqs 1 and 3 leads to the connection between surface coverage Γ and the single-surfactant concentration *c*_1_ in the bulk
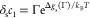
8Here, Δ*g*_s_(Γ) = μ_s_^ex^(Γ) – μ_w_^ex^ is the difference between the surfactant excess chemical potentials in the monolayer and in the bulk water phase. It hence represents the transfer free energy for bringing a free surfactant from bulk water to the interface. The transfer free energies Δ*g*_s_(Γ) will be computed by the same procedure as Δ*g*_m_^(*n*)^ for micelles. Evaluating both kinds of free energies is the core computational challenge of this work, as explained in Section 3 further below.

There is also another, computationally less demanding, approach to compute Δ*g*_s_(Γ), benefiting from known interfacial pressures Π at given surface coverages Γ, that is, the EoS. The starting point of this approach is the Gibbs adsorption isotherm, dγ = −Γdμ_s_, or dΠ = Γdμ_s_ when expressed in terms of the surface pressure. The chemical potential can then be written as the integral μ_s_ = *∫*Γ^–1^dΠ and further decomposed into the ideal and excess parts using eq 3, bringing us to^11,22,27^
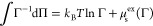
9Note that we have dropped constant terms, which we will determine at the very end. To switch the integration variable to Γ, we perform the integration *per partes* of the left-hand side, *∫*Γ^–1^dΠ = Γ^–1^Π – *∫*ΠdΓ^–1^, and obtain
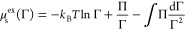
10We now express the excess chemical potential μ_s_^ex^ with the transfer free energy Δ*g*_s_(Γ) = μ_s_^ex^(Γ) – μ_w_^ex^ and set the integration boundaries ranging from 0 to a finite coverage Γ. Finally, the integration constant is set by the reference value Δ*g*_s_(0) at zero coverage Γ = 0, which finally gives us
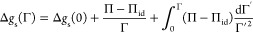
11Here, Π_id_ = *k*_B_*T* Γ is the surface pressure in the ideal-gas limit. The basis of this approach is the integration of the EoS, and we will refer to it as the Π*-integration* method. Obviously, this method allows obtaining only the increment of the transfer free energy from a reference point—zero coverage in our case. The reference has to be determined in another way, as described in the following section.

## 3 Simulation Methods

### 3.1 Simulation Details

We employed the GROMOS-compatible 2016H66 force field^28,29^ for the simulation of C_12_EO_6_ surfactants, which adopts a united-atom representation of aliphatic groups. This force field, which was optimized independently for pure-liquid as well as polar and nonpolar solvation properties of small organic molecules, was also shown to represent a good starting point for simulating C_*n*_EO_*m*_ surfactants.^29^ The alkane phase consisted of *n*-decane, simulated by the standard united-atom GROMOS force field.^30^ Water was simulated by the SPC/E model.^31^

Spherical micelles were simulated in cubic boxes of edge length 6.8 nm using the isotropic pressure coupling. Micelles adopted the spherical shape naturally after a nanosecond of equilibration from a preformed aggregate. The decane/water simulation setup consisted of 3–4 nm thick water and decane slabs in close contact in a simulation box of dimensions around 5 nm × 5 nm × 10 nm (shown Figure 2B). The system was replicated in all three directions via periodic boundary conditions, giving us two decane/water interfaces in the box. Both interfaces were covered with the same number of surfactants to form monolayers. Various surfactant coverages Γ were realized by symmetrically varying the number of surfactants on both interfaces while leaving the lateral box dimensions fixed. The atmospheric pressure was maintained by adjusting the box dimension perpendicular to the interfaces. The simulations were performed with the GROMACS 2019.4 simulation package.^32^ The Lennard-Jones (LJ) interactions were truncated at 1.4 nm. Electrostatic interactions were treated with Particle-Mesh-Ewald methods^33,34^ with a 1.4 nm real-space cutoff. The temperature in all simulations was set to 300 K using the velocity-rescaling thermostat^35^ with a time constant of 0.1 ps. The atmospheric pressure was maintained using the Parrinello–Rahman barostat^36^ with a time constant of 1 ps.

### 3.2 Thermodynamic Integration (TI)

The transfer free energies of surfactants into micelles, Δ*g*_m_^(*n*)^, and onto the interface, Δ*g*_s_, were computed using the thermodynamic integration (TI) technique^37^ with the surfactant interaction potential as the thermodynamic path. In this procedure, we chose one surfactant molecule as the *test particle* whose partial charges and LJ interactions were gradually faded out. This was done by introducing a coupling parameter, λ ∈ [0, 1], that continuously switches the interactions in the Hamiltonian *U*(λ) between the fully interacting (λ = 1) and a noninteracting (λ = 0) surfactant. The surfactant’s excess chemical potential (or *solvation free energy*) for this “alchemical” transformation process is computed as
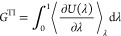
12The transfer free energy for bringing a surfactant from the bulk water phase into the aggregate (a micelle or monolayer) was computed as the difference between the solvation free energies in the aggregate and in bulk water, Δ*g*_agg_ = *G*_agg_^TI^ – *G*_w_^TI^.

In order to facilitate numerical convergence, the test surfactant in an aggregate was gently restrained with harmonic potentials at two locations to fixed positions in space during the TI procedure, namely: (i) at the second oxygen atom from the hydroxide terminus and (ii) at the third carbon atom from the alkyl terminus. The used spring constants in each spatial dimension are given in Table 1. The purpose of the restraints is to suppress undesired translational and rotational motions, which could lead to poor sampling. The conformational configurations of the fully interacting surfactant are similarly restricted by the aggregate, therefore, the applied restraints are not expected to significantly suppress the entropic contribution to the free energy. For the TI of a free surfactant in bulk water, no restraints were applied, as in that case, they are not needed and could influence the conformational configurations. We also restrained all other (unperturbed) surfactants in the micelle but with much weaker potentials (see Table 1) to suppress translation, rotation, and large-scale shape deformations of the micelle during TI.

**Table 1:** Harmonic Restraining Potentials (given in kJ mol^–1^ nm^–2^) in Different Directions Applied to the Test Surfactant and Other Surfactants in Different Settings

		*k*_*x*_	*k*_*y*_	*k*_*z*_
Bulk water	Test surfactant	0	0	0
Alkane/water interface	Test surfactant	0	0	50
	Other surfactants	0	0	0
Spherical micelle	Test surfactant	50	50	50
	Other surfactants	5	5	5
Cylindrical micelle	Test surfactant	50	50	0
	Other surfactants	5	5	0

The TI (eq 12) was performed in two steps: At first the partial charges of the test surfactant were linearly scaled down while keeping the LJ interactions unaltered. In the second step, also the LJ interactions were scaled down using the “soft-core” LJ functions provided in GROMACS to bypass the singularities near the vanishing potential strength (λ → 0).

The Coulomb part of the TI procedure was divided into 24 individual simulations with equidistant λ values between 0 and 1. Likewise, 24 λ values were used for the LJ part, which are denser in the interval λ = 0.3–0.5, where the derivative ⟨*∂U*/*∂λ*⟩ exhibits pronounced variations (see the SI). These variations are due to abrupt jumps of a moderately interacting test surfactant with its neighbors. Each λ simulation is 4 ns long, from which the first 0.2 ns were discarded from sampling to ensure equilibrium. The derivative *∂U*/*∂λ* was computed every picosecond.

Finally, we have to point out that there are alternative approaches for calculating transfer free energies, which could also be used for our problem. Arguably the most popular are umbrella-sampling simulations,^11,23,24,38^ which may be more straightforward to implement, but have certain disadvantages. For instance, pulling the test surfactant out of a deformable micelle or interface gives rise to local distortions that can lead to poor convergence. Moreover, in contrast to pulling, TI does not rely on a physical path between the two phases and can be used in more complex situations like highly concentrated micellar phases or bicontinuous microemulsions.

## 4 Results and Discussion

### 4.1 Free Surfactants

Before venturing into the complexity of aggregate formation and interface adsorption, we have to justify the treatment of the aqueous phase of free surfactants as an ideal solution, which assumes a concentration-independent excess chemical potential μ_w_^ex^ in eq 1. The validity of the ideal regime can be readily estimated on the second-order virial expansion level. As described in the SI, a simulation of two surfactants in water was used to evaluate the second virial coefficient. With the obtained value of *B*_2_^3D^ = −0.1(3) nm^3^ we expect that deviations from ideality kick in for concentrations approaching  M, which is several orders of magnitude above the CMC of C_12_EO_6_ surfactants. This means that interactions between free surfactants in the water phase can be neglected for all considered concentrations and that μ_w_^ex^ can be considered as independent of *c*_1_, as assumed in eq 1.

### 4.2 Micelle Formation

**Figure 3:**
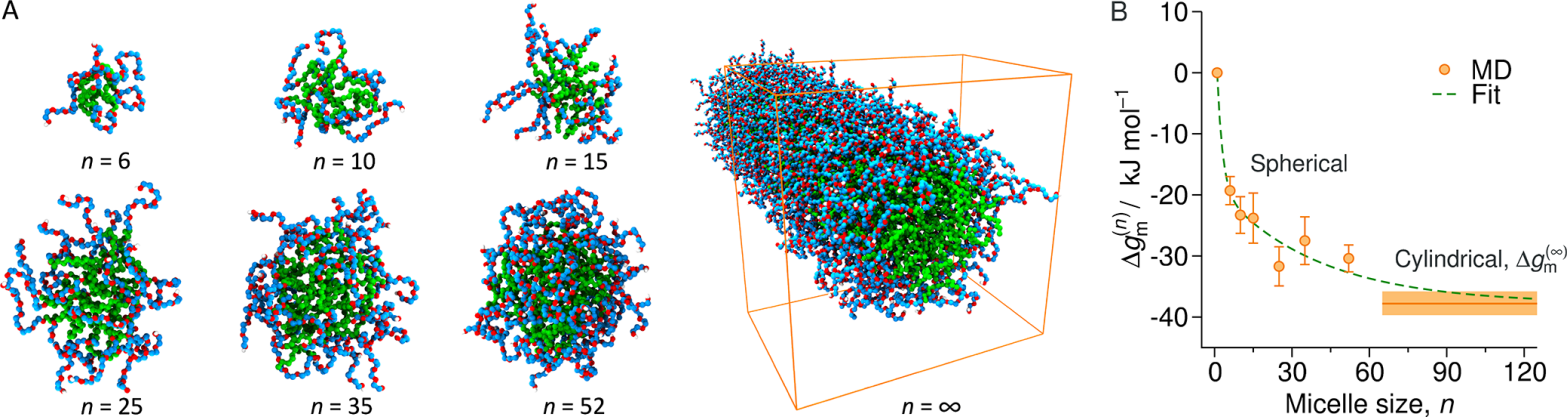
(A) Snapshots of the simulated micelles of different sizes *n*, including an infinitely long cylindrical micelle (*n* = *∞*). (B) Transfer free energy of a surfactant into a micelle of final size *n*. The dashed green line is a double-exponential fit that saturates at Δ*g*_m_^(*∞*)^.

To examine the micellar equilibrium, we simulated micelles of various sizes in bulk water, shown in Figure 3A. In addition, we carried out a simulation of a cylindrical micelle replicated across periodic boundary conditions in the longitudinal direction, mimicking an infinitely long cylindrical micelle. This micelle was equilibrated in the semi-isotropic ensemble at 1 bar so that the internal tension vanished. The resulting linear number density of surfactants was d*N*/d*l* = 27 nm^–1^, and its effective radius, based on the partial molar volume, is R* = 2.55 nm. The effective surfactant volume is then *v* = πR*^2^(d*l*/d*N*) ≈ 0.76 nm^3^, which is very similar to the partial molar volume of one free surfactant in water, *v* = 0.77 nm^3^, obtained from an independent simulation.

Figure 3B shows the transfer free energies Δ*g*_m_^(*n*)^ for bringing a surfactant into the micelle of size *n*. The values were obtained with the TI approach described in Section 3 and monotonically decrease with micelle size. They asymptotically approach the value for the cylindrical micelle of Δ*g*_m_^(*∞*)^ = −38(2) kJ/mol, indicated by the solid horizontal line. The asymptotic behavior implies that worm-like, cylindrical micelles are thermodynamically favorable over spherical ones and will eventually overpopulate the system at high total concentrations. Indeed, this theoretical prediction by the model is also supported by experiments for C_*n*_EO_*m*_ surfactants.^39−41^

Before turning into computing micelle concentrations with eq 6, let us examine under which conditions this iterative scheme converges. The convergence is met if *c*_*n*_ < *c*_*n*–1_ for *n* → *∞*, which is fulfilled for concentrations *c*_1_ < *c*_1_^CMC^. The critical value *c*_1_^CMC^ matches the limiting case of *c*_*n*_ = *c*_*n*–1_, which follows from eq 6 as
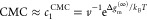
13As we will elaborate further below, *c*_1_^CMC^ corresponds to the condition at the CMC. Moreover, it is to an excellent approximation equal to the CMC, hence *c*_1_^CMC^ ≈ CMC. The estimate for the CMC given by eq 13 amounts to CMC ≈ 5_–3_^+6^ × 10^–7^ M from our simulation data, a result which will be compared with the experimental value further below.

**Figure 4:**
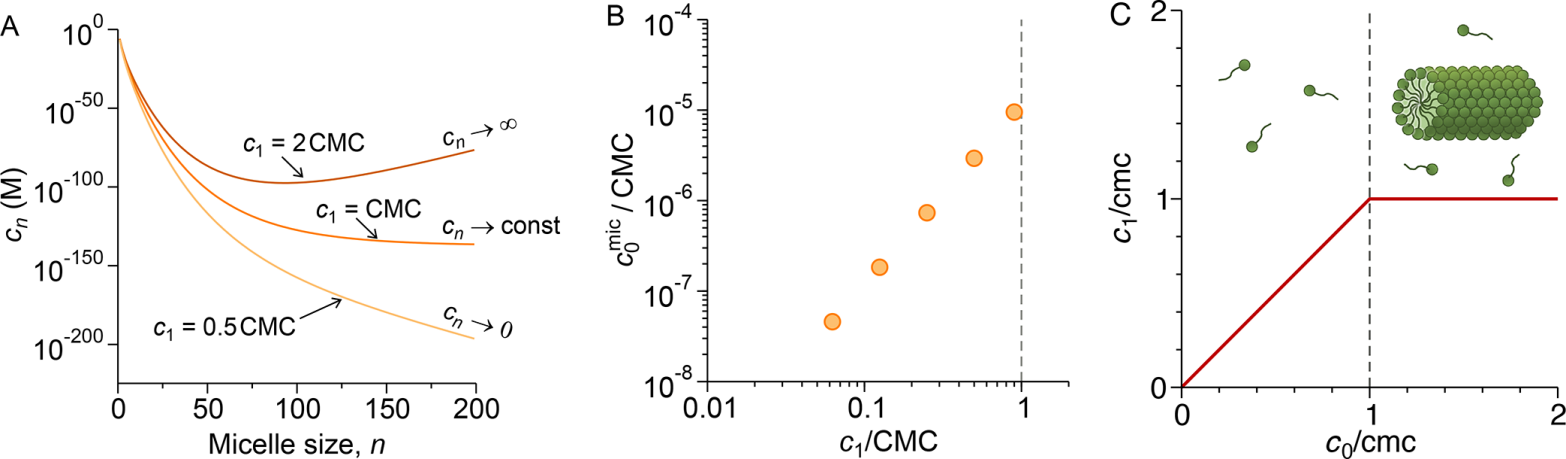
(A) Calculated micelle concentrations for different single-surfactant concentrations *c*_1_ (using eq 6). (B) Total concentration of surfactants inside micelles (with *n* ≥ 2) versus single-surfactant concentration *c*_1_. (C) Single-surfactant concentration versus total concentration rescaled by the CMC (calculated from eq 14). Below the CMC, surfactants are primarily free. Above the CMC, the surplus of surfactants goes into forming long cylindrical micelles.

The iterative scheme (eq 6) for computing the micelle-size distribution requires Δ*g*_m_^(*n*)^ for all micelle sizes *n*, which we did not evaluate. To circumvent the problem of missing data points, we perform an empirical fit to the MD data points in Figure 3B (dashed green line) with a double-exponential function of the form Δ*g*_m_^(*n*)^ = Δ*g*_m_^(∞)^[1 − *A*e^−α_1_(*n*−1)^ − (1 − *A*)e^−α_2_(*n*−1)^]. The data points are reproduced well for *A* = 0.5, α_1_ = 0.48, and α_2_ = 0.026. Using this fit with eq 6, we finally compute micelle-size distributions for three single-surfactant concentrations, *c*_1_, plotted in Figure 4A. Below the CMC, the micelle population exponentially decays with size, whereas it saturates for *c*_1_ = CMC. For illustration, we also show a hypothetical scenario of *c*_1_ = 2 CMC, for which concentrations would exponentially diverge with micelle size. The meaningful physical interpretation of the latter behavior is that once the total concentration *c*_0_ surpasses the CMC, concentrations of free surfactants and smaller-size micelles remain fixed, whereas the remaining surfactants go into the formation of long cylindrical micelles.

It is insightful to assess the contribution of micelles (characterized by *n* ≥ 2) to the total concentration *c*_0_ in terms of eq 7. The micellar contribution, *c*_0_^mic^ ≡ *c*_0_ − *c*_1_ = ∑_*n*=2_^∞^*nc*_*n*_, is plotted in Figure 4B as a function of *c*_1_. We see that on the entire range below the CMC, the micellar contribution is orders of magnitude below the single-surfactant contribution, *c*_0_^mic^ ≪ *c*_1_, merely reaching *c*_0_^mic^/*c*_0_ ≈ 10^–5^ at the CMC. We thus conclude that below the CMC, the solution is essentially composed of individual free surfactants with a negligible population of micelles, hence *c*_0_ ≈ *c*_1_. Above the CMC, *c*_1_ remains fixed at the CMC, and the surplus of surfactants is consumed by long cylindrical micelles. This consideration allows us to construct the following relation between *c*_1_ and *c*_0_,
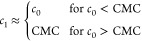
14as plotted in Figure 4C. That is to say, a single phase of free surfactants exists below the CMC, whereas above the CMC, a coexistence between free surfactants and cylindrical micelles is established. Note that the free surfactant concentration remains constant above the CMC only as long as individual micelles do not interact with one another, which only occurs at very high concentrations.^42^

### 4.3 Adsorption at the Alkane/Water Interface

We start investigating the alkane/water interface with the EoS of the surfactants, Π(Γ), which can be obtained relatively easily in simulations. The adsorption Γ is a control parameter in our simulations, and the surface pressure Π is the response, computed from resulting surface tensions^43^ as Π = γ_0_ – γ, where γ is the surface tension of a finite coverage and γ_0_ = 54.1(4) mN/m is the surface tension of the bare decane/water interface in our model.

**Figure 5:**
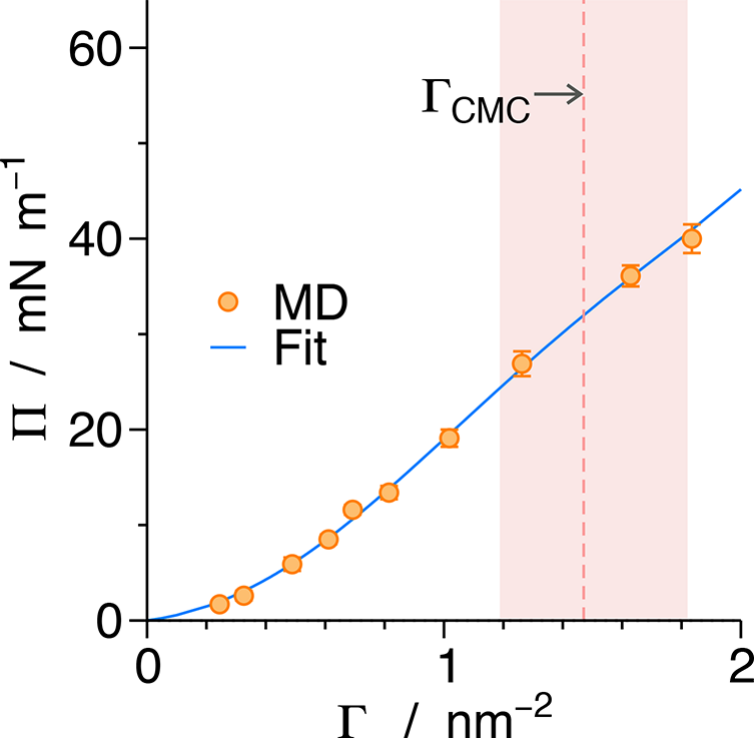
Equation of state: Surface pressure versus excess adsorption (orange circles). The blue solid line is a fifth-order polynomial fit to the MD data points (see the text). The red-shaded region is the *a posteriori* predicted adsorption at the CMC.

The evaluated EoS is shown in Figure 5 by circle symbols. The MD data points are fitted with the polynomial Π(Γ) = *k*_B_*T*Γ + *C*_2_Γ^2^ + ... + *C*_5_Γ^5^, where the first term corresponds to the ideal 2D gas surface pressure, whereas the higher-order terms capture the deviation from ideality, with the coefficients *C*_2_, ..., *C*_5_ used as fitting parameters. At this point, we recall that the EoS cannot be compared directly to experiments of this surfactant category owing to challenging direct measurements of Γ^44^ and to the rapid exchange of surfactants between interface and bulk, which prohibits indirect estimates of Γ.

**Figure 6:**
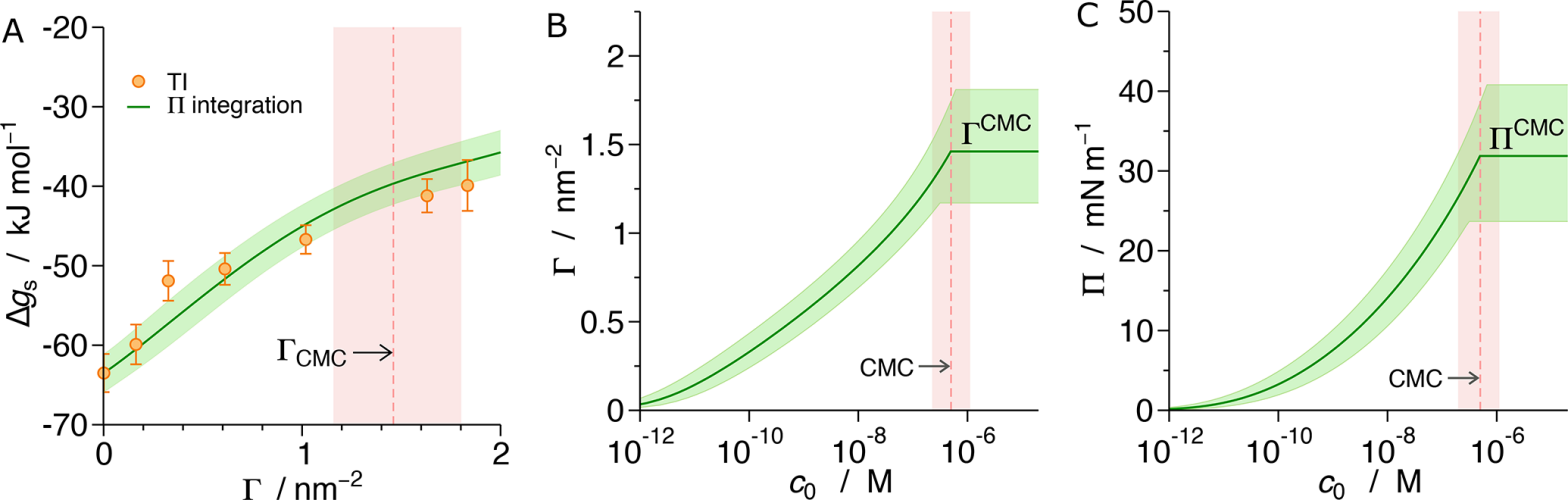
(A) Transfer free energy of a surfactant from bulk water to the alkane/water interface as a function of surface coverage. Symbols are MD data computed using the TI method. The green line is the integrated Π(Γ) curve using eq 11, and the green-shaded area represents its uncertainty. (B) Computed adsorption isotherm from eqs 8 and 14. (C) Pressure isotherm obtained from panel B by exchanging Γ for Π using the equation of state (Figure 5). The red shaded areas in all three panels indicate values at the CMC.

We therefore move on to free-energy calculations. Using the TI procedure described in Section 3, we computed transfer free energies of surfactants to the interface at various coverages Γ, which are shown in Figure 6A as orange circles. In addition to the TI method, we also used the Π-integration approach given by eq 11 in which we used the fitted function Π(Γ). The reference value in the limit of vanishing coverage, Δ*g*_s_(0) = −63.5(2.4) kJ/mol, is taken from the TI procedure, such that both methods coincide at Γ = 0. Both methods match very well within numerical accuracy. Even though they both are mathematically exact, the agreement reassures their correct technical implementations and accurate numerical evaluations. On the one hand, the Π-integration method is computationally much less intensive than the TI method. On the other hand, an advantage of the TI method is its generality, which can be used in circumstances where the surface tension cannot be evaluated, for instance, for surfactant adsorption onto solid surfaces.

Overall, the transfer free energy Δ*g*_s_(Γ) monotonically rises with Γ, meaning that it gets less and less favorable to adsorb additional surfactants when the interface is increasingly loaded with surfactants. Such a behavior stems from an overall repulsive interaction between adsorbed surfactants at the interface, which is also reflected in a positive two-dimensional second virial coefficient at the interface (see the SI). The fact that surfactants effectively repel each other keeps the monolayer uniform and prevents the formation of in-plane clusters.^21^

With the obtained free energies, we can now calculate the adsorption Γ at a given total bulk concentration *c*_0_. We do this by combining eq 8 (which relates Γ and *c*_1_) with eq 14 (which relates *c*_1_ and *c*_0_). The thickness δ_s_ in eq 8 is the length that the surfactant atoms can explore in the *z*-direction, which can be estimated from the density distributions of central surfactant atoms as δ_s_ ≈ 0.5 nm (see the SI). In this calculation, we used the free energy values Δ*g*_s_(Γ) obtained with the Π-integration method. The resulting adsorption isotherm, Γ(*c*_0_), is plotted in Figure 6B as a solid green line. Γ monotonically rises with *c*_0_ all the way up to *c*_0_ = CMC (indicated by a red stripe), where it reaches Γ_CMC_ ≈ 1.5(3) nm^–2^, which corresponds to the area per surfactant Γ_CMC_^–1^ ≈ 0.7(1) nm^2^. Above the CMC, a coexistence of free surfactants and micelles in the bulk solution is established and the chemical potential (as well as *c*_1_) no longer rises with further increase in total concentration. Consequently, the interfacial coverage stays at the value Γ_CMC_ that has been reached at the CMC.

Furthermore, we can now combine Γ(*c*_0_) and Π(Γ) to eliminate Γ and, in this way, finally construct the pressure isotherm, Π(Γ(*c*_0_)) → Π(*c*_0_), which is shown in Figure 6C. The resulting Π(*c*_0_) behaves qualitatively the same as the adsorption isotherm: It monotonically rises with the total concentration up to the CMC, above which it remains constant at Π_CMC_ ≈ 32(8) mN/m. The pressure isotherm in Figure 6C represents the central result of our modeling procedure. Importantly, pressure isotherms are experimentally easily accessible, whereas the availability of reliable experimental data in the form of surfactant adsorption isotherms or EoS is still very limited.^44^

At first glance, the presented approach may appear numerically costly. However, the computational effort can be reduced significantly with the Π-integration method, and only a few TIs are required for validation.

### 4.4 Comparison with Experiments

**Figure 7:**
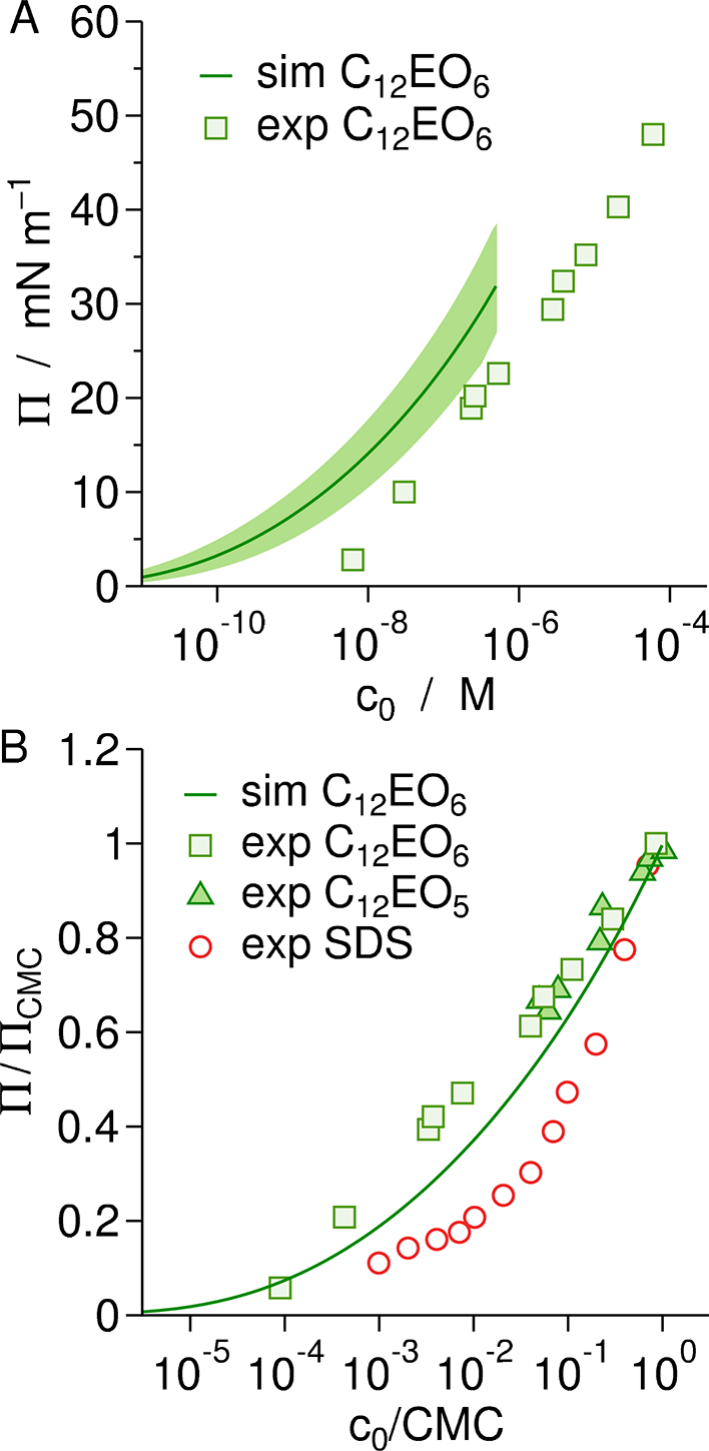
Comparison of simulated pressure isotherm for C_12_EO_6_ from this work to experiments on (A) the absolute scale and (B) reduced scale. Experiments include C_12_EO_6_ at decane/water interfaces,^45^ C_12_EO_5_ at hexane/water interfaces,^49^ and the anionic surfactant SDS at the dodecane/water interface.^53^

In Figure 7, we compare our pressure isotherm with an experimental one by Mulqueen and Blankschtein^45^ for C_12_EO_6_ at the decane/water interface. Both data sets are plotted up to their respective CMC values. We notice that the curves are similar but systematically shifted on the concentration axis. This shift is consistent with a shift in the CMC: The experimentally measured CMC is 7–9 × 10^–5^ M,^46−48^ which is about 2 orders of magnitude higher than our MD-based result. This inconsistency apparently arises from an overestimation of the affinity of surfactants to the micelle Δ*g*_m_^(*∞*)^ in the used computer model, which enters the exponent of eq 13. The underprediction of the CMC is a general problem of the atomistic models currently available,^42^ as we will discuss in Section 4.5. The mismatch between the simulation and experimental values of the free energies follows from eq 13 as
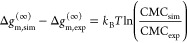
15where we assumed the surfactant volume *v* in the simulations matches the experimental one. The difference amounts to −12(2) kJ/mol (i.e., – 5(1) *k*_B_*T*), meaning that the simulation model overestimates the affinity of surfactants to micelles by about 50%. With this, the validation of the CMC is, at the same time, a validation of the free energy contributions driving self-assembly. An inaccurate CMC value indicates an inaccurate driving force, which affects the aggregation properties, such as their stability and susceptibility to other compounds.^2^

A way to eliminate inevitable systematic discrepancies in the CMC due to imperfect force fields is to compare the results with experiments in such a way that the absolute values of Δ*g*_m_^(*∞*)^ and Δ*g*_s_ drop out of the respective equations. For instance, if we subtract eq 8 at the CMC condition from itself at a general concentration below the CMC, where *c*_1_ = *c*_0_, we obtain
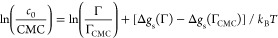
16The difference between the adsorption free energies in the second term on the right-hand side can be expressed in terms of surface pressure by applying eq 11 as
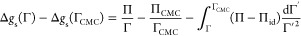
17It can be seen that the relative concentration *c*_0_/CMC is only related to the EoS, Π(Γ), but unrelated to the adsorption free energy. In other words, *c*_0_/CMC is related only to the in-plane interactions between surfactants at the interface. A meaningful validation of the EoS is thus to compare simulation data with experimental ones in terms of the reduced quantities Π/Π_CMC_ and *c*_0_/CMC, which we plot in Figure 7B. In the same graph, we also include data by Aveyard et al.^49^ for C_12_EO_5_ at the hexane/water interface. In that work, the surface tension is presented as a function of the surfactant concentration in the oil phase, *c*_oil_. We have therefore reconstructed the surfactant concentration in the aqueous phase as *c*_0_ = *c*_oil_/*K*_p_, where *K*_p_ ≈ 45 is the partition coefficient of C_12_EO_6_ between decane and water.^50^ The data points from the two experimental systems are virtually identical in the high concentration regime, which reflects that C_12_EO_*m*_ and C_12_EO_*m*+1_ have a very similar behavior^51^ and that the length of the oil molecules is of minor importance, as was noted before.^52^

Our simulation data (solid line in Figure 7) are in good agreement with both experiments at sufficiently high concentrations, but they deviate from the experimental data points for C_12_EO_6_ at lower concentrations. This systematic deviation must be attributed to deviations in Π_CMC_, which in their work is reported as Π_CMC_ ≈ 48 mN/m, whereas we obtained Π_CMC_ = 32(8) mN/m. At this point, it should be noted that Mulqueen and Blankschtein^45^ used the less common ring technique for their measurements, such that some care should be taken when absolute values are compared. In any case, we find that the simulation data are in overall convincing agreement with experimental data on C_12_EO_*m*_ at oil/water interfaces, particularly regarding the shape of the curve on approach to the CMC. For the sake of comparison with different surfactant chemistries, we also included in the plot experimental data from an anionic surfactant (sodium dodecyl sulfate, SDS) at a dodecane/water interface.^53^ It can be seen that Π/Π_CMC_–vs–*c*_0_/CMC curves are not universal but surfactant-specific. The MD-based prediction agrees very well with the C_12_EO_6_ curve but not with that of SDS, which demonstrates that the simulations properly represent the chemical details.

### 4.5 Force Field Limitations

As can be seen in Figure 7, the agreement of the simulation results with experiments is only semiquantitative. As a result of significantly overestimated micelle formation and adsorption free energies, the simulation model underestimates the CMC and consistently exhibits a shift in the effective concentration scale for adsorption (see Figure 7A). The employed force field is, thus, the limiting factor in the present work. An immediate question is whether other available force fields would perform better. Certain estimates can be made based on the existing literature: Huston and Larson^22^ used the GROMOS 53a6_OXY+D_ force field to compute the adsorption of C_12_EO_2_ and C_12_EO_8_ onto a hexadecane/water interface with the biased umbrella pulling method. The interpolation to C_12_EO_6_ gives the adsorption free energy of −62 kJ/mol, which matches our result. In the study by Luz et al.,^23^ the combination of a general Amber force field (GAFF) and SPC/E water also seems to suggest too strong affinity of C_8_EO_*m*_ to a heptane/water interface, which correlates with a too low CMC, as discussed above. Yuan et al.^54^ computed the escape time for C_12_EO_5_ surfactants from a micelle of size *n* = 54 employing the CHARMM force field and TIP3P water. They obtained Δ*g*_m_^(54)^ ≈ – 30 kJ/mol, which is very comparable to our result shown in Figure 3C. Therefore, we also expect the CHARMM/TIP3P combination to yield very similar results for the CMC to ours. All these estimates suggest that currently available atomistic force fields systematically yield too strong adsorption affinities and underpredict experimental CMCs, as already realized by Jusufi and Panagiotopoulos,^42^ who pointed to the need for the development of improved models.

### 4.6 Adsorption to the Air/Water Interface

As motivated in the beginning, this study is focused on C_12_EO_6_ at alkane/water interfaces. Nevertheless, we also determined the adsorption free energy of this surfactant for the air/water interface^41^ in the limit of vanishing coverage, Γ = 0. Strikingly, the obtained value from the TI method, Δ*g*_s_(air/water) = −33(2) kJ/mol, is smaller in magnitude by almost a factor of 2 compared to that from the alkane/water interface, Δ*g*_s_(alkane/water) = −63(2) kJ/mol. This difference, δ*g*_s_ = Δ*g*_s_(air/water) – Δ*g*_s_(alkane/water), must be mainly attributed to the favorable dispersion interactions between the surfactant tails and the alkane molecules.^5^ Consequently, surfactant adsorption to alkane/water interfaces sets in at much smaller bulk concentrations than to air/water interfaces. The shift in the equivalent concentration scale is exp(δ*g*/*k*_B_*T*) ≈ 6  × 10^–6^. Such a shift, together with the differences between the EoS at the air/water and alkane/water interfaces that have been discussed earlier for ionic surfactants,^21^ can be considered key to understanding the experimentally well-described different action of surfactants at air/water and alkane/water interfaces.^53,55,56^ In fact, a shift by ≈5 orders of magnitude in the limit of low coverage, where Π ≈ *k*_B_*T* Γ is independent of the interface type, is in rather good agreement with the Π(*c*_0_) data in these experimental studies. Such considerations will be most rewarding for surfactants for which reliable experimental data at air/water and oil/water interfaces are available.

## 5 Conclusions

Molecular simulations accounting for micellization and adsorption equilibria are a promising way to deepen our mechanistic understanding of the action of surfactants. Yet, the adsorption and self-assembly of surfactants remain a modeling challenge because atomistic MD simulations cannot always capture the relevant time scales. We have combined advanced simulation techniques with a self-consistent theoretical framework that enabled us to obtain a full thermodynamic description of micellization and interface adsorption. The method yields a comprehensive description of the equilibrium among free surfactants, surfactants in micelles, and surfactants at the interface. With this, one can finally disentangle the mechanisms affecting the adsorption isotherm Γ(*c*_0_) and mechanisms affecting the equation of state Π(Γ). Both manifest in the experimental pressure isotherm, Π(*c*_0_), such that a mechanistic interpretation of experimental data without simulations is often difficult. Our analysis revealed that simulation force fields are currently the limiting factor for a quantitative comparison with experiment—an obstacle that must be urgently overcome. In this regard, our approach is also suited for surfactant force field validation. Logical and crucial extensions of the current framework in future research are applications to (a) nonionic surfactants at water/air interfaces and (b) ionic surfactants at both alkane/air and water/air interfaces. Ionic surfactants introduce a collective long-ranged electrostatic interplay with counterions and co-ions, resulting in a more complex and nonlinear adsorption behavior.^3,21,57^ While the TI method lends itself also to computing the free energies of ionic surfactants, the theoretical thermodynamic framework in this case may have to be extended to include long-range electrostatic effects.

## Data Availability

Simulation files are openly available.^58^

## Supporting Information Available

Additional details on Thermodynamic Integration, Monolayer thickness, Second virial coefficients (PDF)
