# Supplementary material for: Interface Adsorption versus Bulk Micellization of Surfactants: Insights from Molecular Simulations
Source: J Chem Theory Comput. 2023 May 22;20(4):1568–78. doi: 10.1021/acs.jctc.3c00223 (PMC10902850; doi:10.1021/acs.jctc.3c00223)
Supplement: Supplementary file 1 — ct3c00223_si_001.pdf [file ct3c00223_si_001.pdf]

# Supporting Information:
# Interface Adsorption versus Bulk Micellization of Surfactants: Insights from Molecular Simulations


Matej Kanduč,[*,†] Cosima Stubenrauch,[‡] Reinhard Miller,[¶] and Emanuel Schneck[¶]

†Jožef Stefan Institute, Jamova 39, 1000 Ljubljana, Slovenia
‡Institute of Physical Chemistry, University of Stuttgart, Pfaffenwaldring 55, 70569 Stuttgart, Germany
¶Department of Physics, Technische Universität Darmstadt, Hochschulstrasse 8, 64289 Darmstadt, Germany

E-mail: matej.kanduc@ijs.si


## 1. THERMODYNAMIC INTEGRATION

We show three examples of the derivative of the interaction potential $\langle \partial U/\partial \lambda \rangle$ in the TI procedure for the Coulomb (Figure S1A) and Lennard-Jones (Figure S1B) parts.

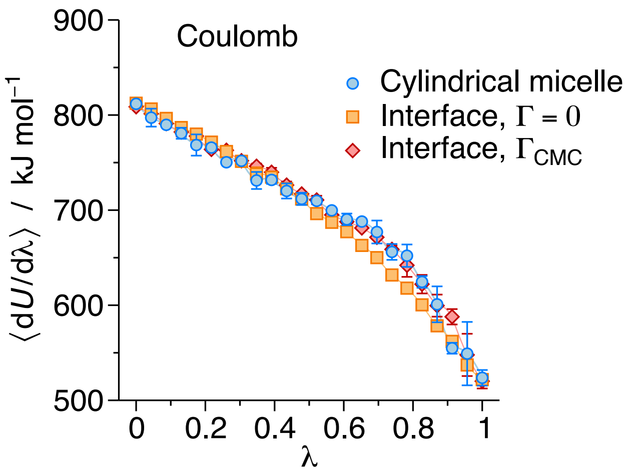


(A)

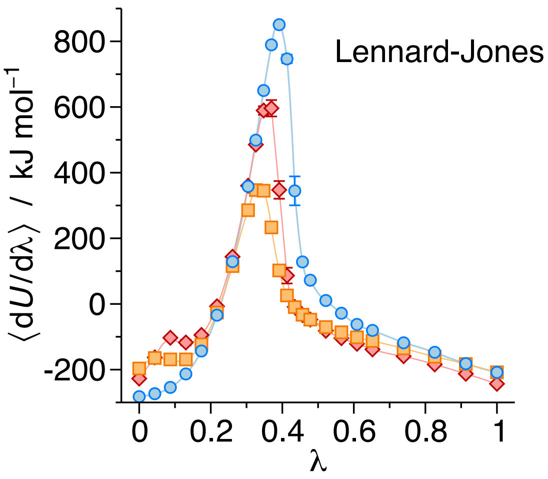


(B)

**Figure S1:** Derivative of the interaction potential with respect to $\lambda$ of the (A) Coulomb and (B) Lennard-Jones part in the TI procedure for three cases: the cylindrical micelle, decane/water interface at zero and full coverage. Symbols are MD data, and solid lines are 2nd-order interpolation splines used for the integration.

## 2. MONOLAYER THICKNESS

Density profiles of the central surfactant atom at the decane/water interface are shown in Figure S2.

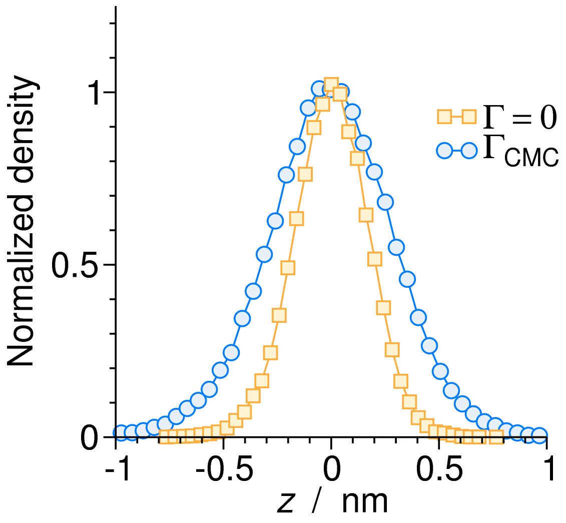


**Figure S2:** Normalized density of the central atom of surfactants across the decane/water interface for two extreme coverages, $\Gamma = 0$ and at the full coverage (at the CMC). The resulting widths of the distribution at half height are $\Delta z_{1/2} = 0.40$ nm and $\Delta z_{1/2} = 0.65$ nm for zero and full coverage, respectively. This suggests using a single value for the monolayer width in our calculations as the mean value, $\delta_\mathrm{s} = 0.5$ nm.

## 3. SECOND VIRIAL COEFFICIENTS

To evaluate the second virial coefficients at the decane/water interface and in bulk, we simulate two surfactants at the interface and in bulk water and evaluate their radial distribution functions ($g_{\mathrm{2D}}$ and $g_{\mathrm{3D}}$, respectively), shown in Figure S3. With these, we compute the 2D second virial coefficient at the interface as

$$B_2^{\mathrm{2D}} = -\pi \int_0^\infty \left[g_{\mathrm{2D}}(r) - 1\right] r \, \mathrm{d}r \tag{S1}$$

which gives $B_2^{\mathrm{2D}} = 2.21(5)$ nm$^2$, implying that the surfactants effectively repel at the interface. The 3D second virial coefficient in bulk is computed as

$$B_2^{\mathrm{3D}} = -2\pi \int_0^\infty \left[g_{\mathrm{3D}}(r) - 1\right] r^2 \, \mathrm{d}r \tag{S2}$$

which results in $B_2^{\mathrm{3D}} = -0.1(3)$ nm$^3$. This value is smaller than the surfactant volume $v = 0.77$ nm$^3$. Hence, two surfactants interact rather weakly in bulk.

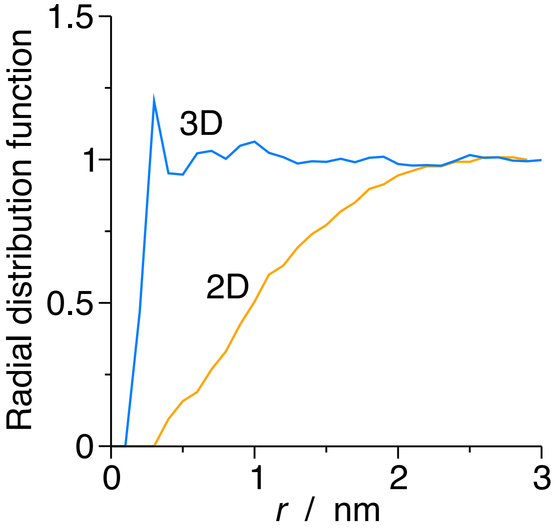


**Figure S3:** Radial distribution functions of two surfactants at the decane–water interface (2D) and in bulk water (3D).
